# (Great) Grandfatherly Advice for Young(er) Dermatologic Surgeons

**DOI:** 10.4103/0974-2077.44172

**Published:** 2008

**Authors:** Lawrence M Field

**Affiliations:** *Inaugural International Traveling Chair of Dermatologic Surgery (International Society for Dermatologic Surgery), University of California, San Francisco (Dermatologic Surgery) (Emeritus 2006), and Stanford University Medical Center, Stanford, California (Emritus 2006) E-mail: lm.fieldmd@comcart.net*

Sir,

Philosophical counseling from their elders is generally not sought by “young dermatologists”. A listing of the “Golden Rules” of practising successful dermatologic surgery has never been proffered. Many younger physicians try to expand their practices by expensive marketing to accomplish successful practices, seemingly unaware of the already existing, prudent, and proper ways adopted by their seniors. In building and maintaining your practices, expensive advertising budgets will never equal the tried and tested basic truths, which may be perhaps slower, but infinitely more sound. This great grandfather seeks to enumerate few of those time-tested tips for his younger colleagues through this letter:

1. Integrity, honesty, and ethics are the cornerstones of success. Financial security follows closely and is critically interrelated.
2. Charge fairly and appropriately. Be charitable towards people, and extend yourself to do charity work.
3. Office aesthetics, waiting room appearance, cleanliness, neatness, washroom area care all impact on your patients and their families as much as your own skills.
4. Be generous with your time and effort in providing patients and their families with the information they need to make intelligent and informed decisions. You will thus, seem not arrogant. “Customer service” is critical.
5. Know how many times your telephone rings before it is answered, and how many are put on hold for extended periods of time – these can identify potential problems.
6. Be prompt with your schedule. To be late does not increase your importance in the patient’s eyes, but may well be interpreted as rudeness and arrogance. If late, apologize, and mean it!
7. Be charitable towards your colleagues. Teach and share with them whenever possible. You will share their good fortune and happiness, and earn their respect and gratitude.
8. Criticise not, that you not be criticised. Professional enemies last forever!
9. Protect the referring doctor from patient criticisms when possible. It will be most deeply appreciated, for most adverse statements return to that same doctor who was being criticized via that same patient or the family.
10. When referred a patient, only do that which was requested of you. Never violate this rule, or be prepared to lose all future referrals by that individual and his/her friends. Emphasise to the patient requesting you to do more, that it is unprofessional to do more than that for which the patient was referred to you.
11. Establish and insist on good, warm, professional, and ethical relationships between your office staff and the staff of your referring physicians.
12. Know what your referred patients and those referring physicians need, and exceed those needs and expectations. Excessive charge complaints are disasters for you.
13. Learn from anyone and everyone who has knowledge, which may benefit your patients. Cross “specialty lines” and professional lines are necessary to accomplish this.
14. Read, study, discuss, practise.
15. Keep formal records of your surgical training and document your learning experiences, in addition to standard CME credits. Photographs and operative reports help.
16. Learn techniques to minimize patient discomfort, both physical and emotional. Extend these considerations to their families.
17. Establish a network of consultants friendly to you, and then do not hesitate to seek consultation when in doubt.
18. Your presentations and publications become your specialty’s means of giving you earned honors and accolades. Be meticulously honest in both. Your intraprofessional reputation depends on it.
19. Get an academic appointment and find the time to teach. You will be rewarded in many ways, as respect from others and from one’s self increase as time passes. Patients love their own physicians to be teachers and givers.
20. Stay alert to the failings of your colleagues. Become an interventionist only via proper professional channels and medical societies. Avoid lay lawyers and media when they seek an opinion on your colleague.
21. Be quick to send your records in response to requests from other physicians. Offer to help in any way you can in the future care of the patient.
22. Express unhappiness with professional nepotism and program control by any self-appointed “chosen few”. Fight it openly and aggressively.
23. Engage in a continuing study of all appropriate journals, going back as far as it is possible to do. Browse your medical centre library monthly if possible, or whenever the opportunity presents itself.
24. Attend appropriate meetings to expand your dermatological surgical knowledge and skills, especially meetings of your local association, the American Society for Dermatologic Surgery, the International Society for Dermatologic Surgery, the American Academy of Cosmetic Surgery, the American Society of Liposuction Surgery, and your local and state dermatologic surgery societies, and, when possible, cross-fertilise with plastic, eye, ear, anaesthesia, etc.
25. Always use/obtain the highest possible quality sutures, staples, equipment, instrumentation, etc. The finest work requires the finest materials.
26. Surgical personalities are generally impatient personalities. Be patient and know yourself by taking the time to obtain and chart histories and/or blood work regarding possible hepatitis, HIV, bleeding dyscrasias, etc. whenever applicable.
27. Answer phone calls from physicians as quickly as possible, and from patients and their families the same day. You should never be too busy for these courtesies. But, in so doing, remember your patient’s rights to privacy when talking to the family!
28. Look and act like a surgeon if you are representing yourself as a dermatologic surgeon. This includes gloves, mask, surgical cap, surgical top, sterile milieu prn, etc. Your surgery room(s) should represent the state of the art in surgical suites, and be reserved for appropriate procedures.
29. Your “comfort level” may, at times, be in competition with or at variance with your “competence level”. At such times, as and when you feel uncomfortable with what you are performing (or about to perform), stop. Wait, get consultation, or refer.
30. Turn down patients whose families are against you doing a particular procedure. That family may never be happy, and can easily set an initially happy patient against you with all the undesirable sequelae.
31. Observe many. Watch, listen, filter. If you must disagree with that which you are observing, politely do so outside the patient’s presence. You should not criticize, and remember, “There are many roads to Rome”, *i.e*., many ways to do things. Experiences and results vary.
32. The more you know, the more you learn – always.
33. Remember who taught you – praise and thank them openly. It will humble you, and remind you of the long path you have taken to knowledge.
34. Forget you have not had complications – it means nothing to the one case in which you do. Approach each as that case.
35. Always err on the side of conservatism, most especially with cosmetic procedures. Law suits last a miserable 3–5 years, or more.
36. Models and body builders seek perfection – even if they deny the same to you. Caution is the word.
37. Ethnic, cultural, religious differences and sensitivities exist between people. Learn, observe, and practise.
38. Never hire a new employee without the acceptance of your previous employees. Jealousy, rancor, dissatisfaction lurk, and office performance suffers.
39. Always remember it is a privilege to practise medicine – practise it both as a science and as an art.
40. Work diligently for respect – both from yourself and from others. Both are hard-earned, and worth every possible effort to achieve!
41. Extend this list by adding your own rules.

These are here for your consideration. And the author hopes and believes that these are as relevant, important, and vital to his younger colleagues as they have been to him.
